# Notes

**Published:** 1884-06

**Authors:** 


TRACTION SUTURE.—Dr. Allis, in the Annals of Anatomy and Surgery, says that when a large portion of integument has been cut away, as in removal of the female breast, the healthy borders sometimes cannot be fully approximated; and even an attempt to do so is accompanied with such a degree of tension that the sutures soon cut their way out. To distribute this tension, after drying the skin thoroughly, he applies strips of adhesive plaster from the margin of the wound in the direction he wishes the sutures to hold. He then passes his needle deeply through plaster and skin. After the sutures are in position, and before tightening them, he requests an assistant to approximate the margins of the wound by pressure from his hands, while he secures them by twisting the wire.—*Louisville Med. News.*

SACRIFICED TO DUTY.—From a foreign exchange we learn that Dr. G. A. Popow, one of the assistant physicians of Prince Peter von Oldenburg's Children's Hospital, while engaged in examining the throat of a bad case of diphtheria, received on his lips and face some of the secretions from the child's mouth. In spite of the most careful cleansing, he was seized next day with shivering, and the first symptoms of an attack of diphtheria, which within five days proved fatal.—*Medical and Surgical Reporter.*

TESTIS IN PERINEO.—Dr. R. L. Macdonnell, in Canada *Medical Record*, August, 1883, relates a case. The patient is fifteen years old. The left testicle has rested in the perineum from the time of his birth. It is situated slightly to the left of the anoscrotal raphe, rather nearer the anus than the scrotum. The organ is well developed, and freely movable. It can be put into its proper place, but cannot be retained there. The scrotum is not so well developed on the left side as upon the right. There is left inguinal congenital hernia. The boy has been under observation for the last five years. He is said to have been born prematurely at the sixth month, and up to the present time has been very delicate, but the deformity has, as yet, caused him no inconvenience.—*American Practitioner.*

---

When you examine the hand of a patient, and find on it the evidence of palmar psoriasis, be that hand the jeweled and perfumed one of a queen, or the dirty paw of a beggar, it is the hand of a syphilitic.—*Hebra.*

CHLORAL FOR HICCOUGH.—The use of chloral as a remedy for obstinate singultus is recommended by Dr. G. C. Kingsbury, in the *British Medical Journal* (Jan. 19, p. 103). A dose of thirty grains is said to have proved sufficient to stop a persistent hiccough with which the patient had suffered incessantly for twelve days.—*Ex.*

CASTOR-OIL AND SCHOOL DISCIPLINE.—According to the *British Medical Journal*, castor-oil is employed as a means of punishment in the West Highland School of Lochgoil-head, Scotland. Breaches of school discipline are treated by "doses of castor-oil" administered, not in the usually prescribed quantity, but by a draught from the bottle. Whatever laxity exists in that school is certainly not on the part of the teachers.—*Ex.*

INFANTICIDE IN FRANCE.—A report has been recently made to the President of the French Republic, in which it appears that in 1882 legal proceedings were taken with respect to 171 cases of infanticide, 19 abortions, and 177 of what is termed the suppression or exposition of infants. The report then goes on to explain that the proportion of acquittals for each of these crimes amounts to 42, 55, and 61 per cent. respectively. As we may be morally certain that nearly every one of the accused was in reality guilty, the enormous proportion of acquittals must seem inexplicable to those who are not intimately acquainted with the conditions of French society. Just, however, as this official report was in course of publication, a seamstress, living in a garret in the Boulevard Voltaire, committed suicide by means of charcoal fumes. The following words were found on the mantelpiece: "I die because I am abandoned at the moment I am about to become a mother." This simple incident explains the statistics we have quoted. The search for proof of parentage is forbidden by the French law, and French juries, knowing that the law gives no protection to betrayed and abandoned women, cannot be made to convict, even where the guilt is patent. *Medical and Surgical Reporter.*

The *Medical Index* is the euphonious title of the consolidated *Kansas City and Missouri Valley Medical Index*, and *The New Medical Era and Sanitarian*, published at Kansas City now. It might bear the motto "E Pluribus Unum," and its conspicuous beginning gives assurance that in union is strength. We trust this new combination may be crowned with success.

---

The remains of the late Dr. S. D. Gross were cremated at Washington, Pa. The ashes weighed about seven pounds. They were hermetically sealed in a tin box, which was inclosed in a marble urn about three feet high, unornamented and without inscription, and placed beside the coffin of his late wife in the family vault at Woodland Cemetery, Philadelphia.

We are indebted to the publisher of the *Southern World* for the cut from which was printed the handsome picture of Dr. A. W. Calhoun, which appeared in the May number of THE JOURNAL.

